# Dose-response analysis of microvasculature changes in the murine fetal brain and the maternal extremities due to prenatal ethanol exposure

**DOI:** 10.1117/1.JBO.25.12.126001

**Published:** 2020-11-26

**Authors:** Raksha Raghunathan, Chih-Hao Liu, Amur Kouka, Manmohan Singh, Rajesh C. Miranda, Kirill V. Larin

**Affiliations:** a University of Houston, Department of Biomedical Engineering, Houston, Texas, United States; b Texas A&M University Health Science Center College of Medicine, Department of Neuroscience and Experimental Therapeutics, Bryan, Texas, United States

**Keywords:** optical coherence tomography, murine embryos, ethanol, brain vasculature

## Abstract

**Significance:** Prenatal exposure to ethanol causes several morphological and neurobehavioral deficits. While there are some studies on the effects of ethanol exposure on blood flow, research focusing on acute changes in the microvasculature is limited.

**Aim:** The first aim of this study was to assess the dose-dependent changes in murine fetal brain microvasculature of developing fetuses in response to maternal alcohol consumption. The second aim was to quantify changes in vasculature occurring concurrently in the mother’s hindlimb and the fetus’s brain after maternal exposure to alcohol.

**Approach:** Correlation mapping optical coherence angiography was used to evaluate the effects of prenatal exposure to different doses of ethanol (3, 1.5, and 0.75  g/kg) on murine fetal brain vasculature *in utero*. Additionally, simultaneous imaging of maternal peripheral vessels and the fetal brain vasculature was performed to assess changes of the vasculature occurring concurrently in response to ethanol consumption.

**Results:** The fetal brain vessel diameters (VDs) decreased by ∼47%, 30%, and 14% in response to ethanol doses of 3, 1.5, and 0.75  g/kg, respectively. However, the mother’s hindlimb VD increased by 63% in response to ethanol at a dose of 3  g/kg.

**Conclusions:** Results showed a dose-dependent reduction in vascular blood flow in fetal brain vessels when the mother was exposed to ethanol, whereas vessels in the maternal hindlimb exhibited concurrent vasodilation.

## 1 Introduction

No amount of alcohol is considered safe for developing fetuses,^1^^–^^3^ but the consumption of alcoholic beverages during pregnancy is still very common.^4^^–^^7^ Prenatal exposure to alcohol in any amount damages the developing fetus and cause congenital defects. In 1973, the stereotypic cluster of the craniofacial, brain, and growth deficits, collectively termed “fetal alcohol syndrome” was identified in infants born to mothers with heavy alcohol exposure during pregnancy.^8^^,^^9^ Since then, research has shown that prenatal exposure to alcohol causes a broad range of developmental defects, which are now termed fetal alcohol spectrum disorders (FASD) that refer to a range of abnormalities caused by prenatal alcohol exposure (PAE).^10^

The estimated global prevalence of FASD is 22.7 per 1000 births, and regional estimates can vary from 33.5 per 1000 births in the United States to 113.22 per 1000 births in South Africa.^11^ In general, 15% of all pregnancies end in spontaneous abortions, but that rate is as high as 45% for women who are heavy drinkers.^12^ Similarly, the number of stillbirths is six-times higher when the fetus is exposed to alcohol as compared to the healthy population.^13^ The severity of the defect or disorder depends on a variety of factors,^14^ but most importantly, the amount of alcohol consumed and the period of gestation during which alcohol was consumed. Other factors that can potentially increase the severity of the defect include concurrent smoking, environmental toxins, and socioeconomic factors.

Due to the prevalence of unplanned pregnancies in the United States^15^ and binge patterns of alcohol consumption,^16^ PAE in the first trimester is common. However, many women continue to consume alcohol well into their second trimester of pregnancy,^17^ which is a consumption pattern that includes the critical periods of both neurogenesis and angiogenesis. The majority of neurons of the adult brain are born during this period.^18^ Although PAE has significant detrimental effects on different organ systems, the central nervous system (CNS) is affected the most due to the extent of its developmental period and sensitivity to ethanol. Thus, to understand the effects of PAE on the developing brain, studying second-trimester exposure to alcohol is crucial. Several aspects of brain development altered by PAE that have been extensively studied include morphological, behavioral, and cognitive effects.^19^^–^^29^ In addition to neurons, the microvasculature in the brain also experiences significant development and growth during the second trimester. The microvasculature supports the nutritional needs of the developing fetus,^30^ provide endocrine control of fetal growth,^31^ and promote neural development.^32^ Although studies have evaluated changes in blood flow after PAE,^33^^,^^34^ acute changes in fetal brain vasculature have not been well documented due to the lack of techniques with sufficient resolution and penetration depth.

Over the past decade, optical coherence tomography (OCT)^35^ has rapidly gained popularity for small animal embryonic imaging.^36^ Due to its noninvasive nature, relatively high spatial and temporal resolutions, and its ability to provide cross-sectional images of live embryos with no exogenous contrast agents, OCT has been preferred over other imaging modalities such as histological staining,^37^^,^^38^ ultrasound biomicroscopy, micro-computed tomography, and micro-magnetic resonance imaging for live imaging of small mammal embryos.^39^ Angiographic OCT is a functional extension of OCT that was developed to image microvasculature.^40^^–^^46^ We have utilized angiographic OCT to assess the effects of prenatal exposure to one dose of ethanol,^47^ a synthetic cannabinoid,^48^ and nicotine on the fetal brain.^49^

This study uses correlation mapping optical coherence angiography (cm-OCA)^50^ to assess dose-dependent fetal brain vasculature changes due to maternal exposure to ethanol in a mouse model *in utero*. Results showed that the severity of vasoconstriction increased with dose. In addition, we also demonstrate assessment of both maternal and fetal vasculature changes due to maternal exposure to ethanol, where peripheral vessels in the mother showed vasodilation in contrast to fetal brain vasculature vasoconstriction.

## 2 Materials and Methods

### 2.1 OCT System

A phase-stabilized swept source OCT system (PhS-SSOCT) was used for angiographic imaging. In summary, the system composed of a broadband swept source laser source of central wavelength 1310 nm, scan rage of 150 nm, scan rate of 30 kHz, output power of 39 mW, axial resolution of 11  μm in air, and lateral resolution of 16  μm. A complete description of the system can be found in our previous publications.^47^^,^^51^

### 2.2 Animal Manipulation and Dosing

Detailed animal manipulation procedures are described in our previous work.^49^ In summary, pregnant CD-1 IGS mice (Crl: CD1{ICR}, Charles River Laboratories, Inc. Wilmington, MA) at gestational day 14.5 were anesthetized and placed on a heated surgical platform to maintain body temperature throughout the imaging procedure. Abdominal fur was removed, and an incision was made on the abdomen exposing the uterine horn for imaging. After stabilization, initial OCT images of the fetal brain were recorded. The mother was then administered the corresponding dose of ethanol via intragastric gavage. Subsequent OCT measurements were taken for a period of 45 min at 5-min intervals. The uterus was hydrated with 1× phosphate-buffered saline 1 min before every measurement.

Our first study evaluating the effects of ethanol on the fetal brain used ethanol at a dose of 3  g/kg^47^ to simulate binge drinking in humans. At this dose, severe vasoconstriction was observed in the fetal brain within 45 min of exposure. Hence, in this study, two lower doses of ethanol, 1.5 and 0.75  g/kg, were used to evaluate the effects of dose on the murine fetal brain vasculature. Sham (control) experiments were performed utilizing distilled water because the ethanol was diluted in distilled water.

There were a total number of six samples in the sham group, three samples in the 3-g/kg ethanol group, four samples in the 1.5  g/kg of ethanol group, and five samples in the 0.75  g/kg of ethanol group. The number of the samples refers to the number of separate fetuses from separate pregnancies. Additionally, three mice were used to study the effect of a dose of 3  g/kg on the maternal peripheral and fetal brain blood vessels simultaneously.

### 2.3 Mother and Fetus Comparisons

Since there is a paucity of research on the acute changes in the developing fetal brain vasculature after maternal ethanol consumption, we simultaneously assessed the effects of maternal alcohol consumption on both the mother and the fetus. Ethanol has different effects on blood vessels based on the location of the vessel in the body, so vessels on the skin of the hind limb of the mother were imaged with the mother in a supine position. This location was chosen because the peripheral vessels on the forearm in humans are known to dilate when exposed to alcohol.^52^ Since the forelimb of the mother was relatively difficult to image due to the surgical procedures on the abdominal cavity, the peripheral vessels on the hindlimb were imaged instead.

A dose of 3  g/kg of ethanol was tested on three mice. Instead of the aforementioned procedure where the fetal brain was imaged every 5 min for 45 min after maternal ethanol consumption, imaging was performed after 30 min to ensure proper imaging of the peripheral blood vessels of the mother and fetal brain. Results from this group were compared to the sham group, where the mother was administered lactated Ringer’s instead of ethanol.

### 2.4 Imaging, Quantifications, and Statistics

The 3D OCT images consisted of B-scans with 600 A-scans each, and a total of 600 B-scans per volume. Five B-scans were recorded at each spatial position to perform angiographic imaging. A total area of ∼6  mm×6.2  mm of the fetal brain was imaged. The total acquisition time for each dataset was 84 s, including scanning mirror flyback time. A correlation mapping cm-OCA algorithm was used to obtain the vasculature maps from the acquired OCT images.^50^ A discrete Fourier transform-based sub-pixel registration technique was used to correct the axial shift caused due to bulk motion between each pair of 5 B-scans that were recorded at the same spatial position.^53^ The temporal correlation for each frame with the reference frame was calculated and averaged. The threshold was then determined as the average of the mean correlation values minus the standard deviations of the mean correlations. The SNR-dependent artifacts were corrected using the temporal variance of the background noise as a function of imaging depth.^50^ The 3D vasculature maps were obtained by mapping the temporal correlation coefficients of the entire 3D image. A maximum intensity projection (MIP) was calculated to obtain the *en face* images of the dorsal surface arterial blood vessels on the fetal brain. A frequency rejection filter^54^ was applied to the 2D MIPs to remove bulk motion artifacts due to maternal respiration and heartbeat. Amira software (EFI Co., Portland, Oregon) was used for denoising and to form final MIPs.

All quantifications were performed on the MIP images, and vessel diameter (VD) was used to quantify the changes in vasculature. Amira was used to perform the quantifications. All VD quantifications were performed on the main branch of the vessel. The results in this study also include images and quantifications from fetuses that were exposed to ethanol at a dose of 3  g/kg from our previously published data.^47^ However, since this study uses a different angiography algorithm, the data were reprocessed using the current cm-OCA algorithm.

For the dose-response study, two nonparametric Kruskal–Wallis ANOVAs were performed to assess the effects of different doses and time on the vasculature. This was followed by two-sided Mann–Whitney U tests that were performed to test for statistically significant changes between every ethanol group and the sham group (three pairs) and between the three ethanol groups (three pairs). Thus, there were a total of six pairwise Mann–Whitney tests that were performed. Bonferroni correction was included in this case for multiple pair-wise tests.

For the mother and fetus comparisons, Mann–Whitney U tests were performed to test for statistically significant changes between the ethanol and sham groups of the mother and between the ethanol and sham groups of the fetuses.

## 3 Results

### 3.1 Dose-Response Analysis

**Fig. 1:**
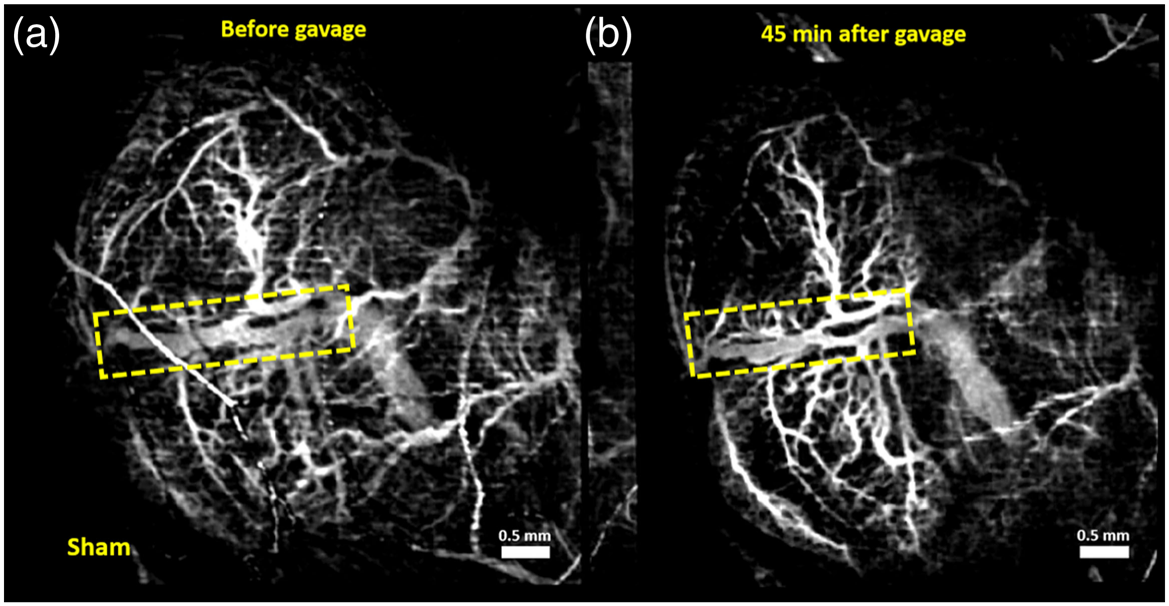
MIPs of cm-OCA images of murine fetal brain vasculature (a) before and (b) 45 min after maternal exposure to distilled water. The dashed rectangle shows the main branch of the vessel on which the quantifications were performed.

**Fig. 2:**
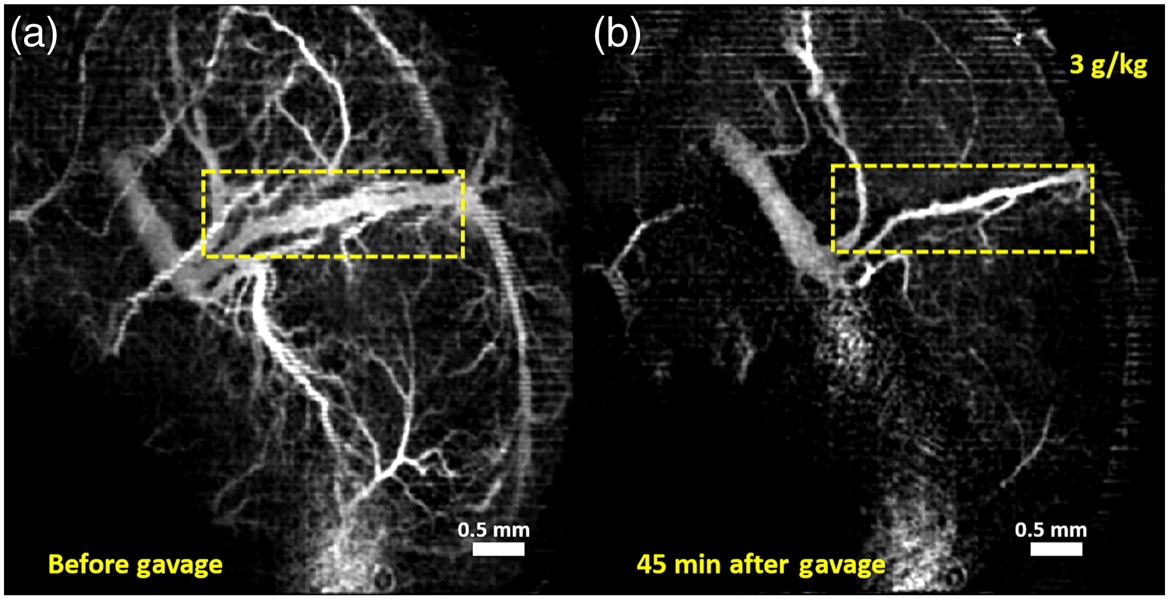
MIPs of cm-OCA images of murine fetal brain vasculature (a) before and (b) 45 min after maternal exposure to ethanol at a dose of 3  g/kg. The dashed rectangle shows the main branch of the vessel on which the quantifications were performed.

**Fig. 3:**
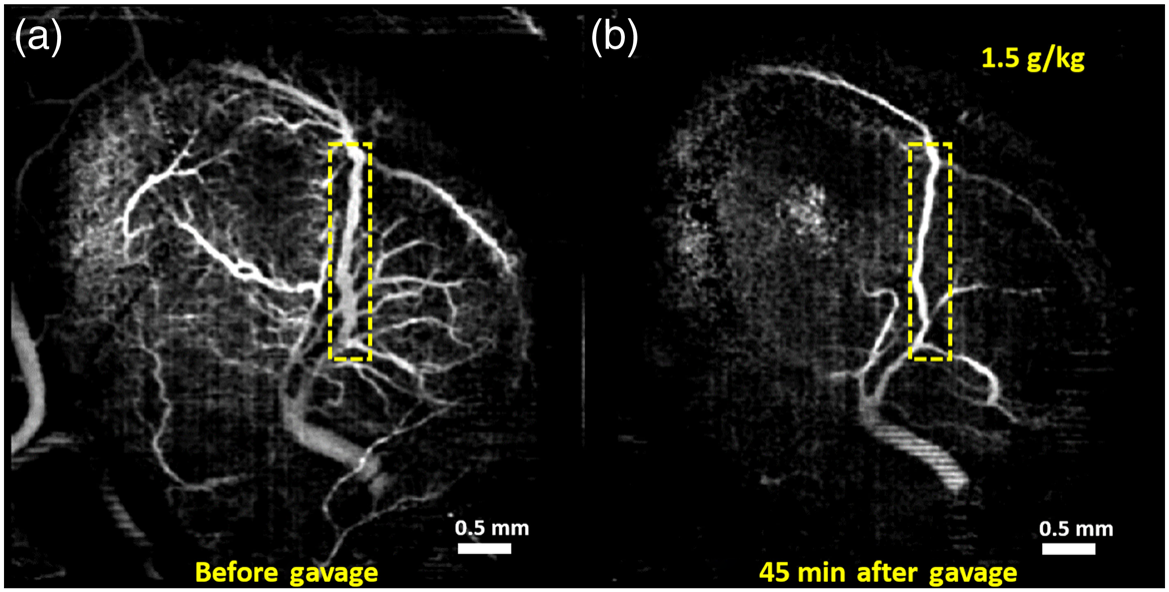
MIPs of cm-OCA images of murine fetal brain vasculature (a) before and (b) 45 min after maternal exposure to ethanol at a dose of 1.5  g/kg. The dashed rectangle shows the main branch of the vessel on which the quantifications were performed.

**Fig. 4:**
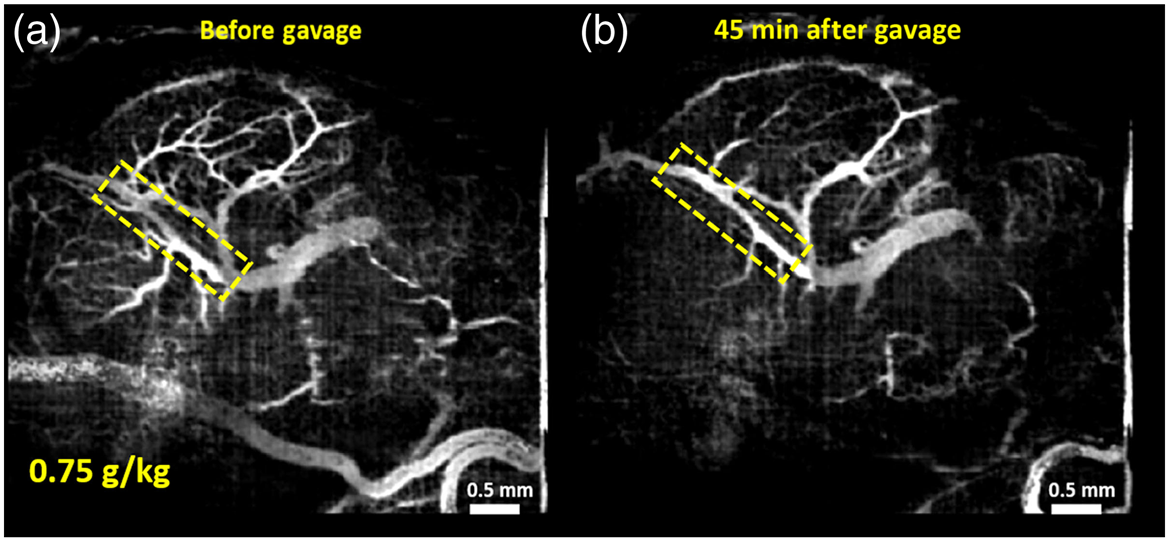
MIPs of cm-OCA images of murine fetal brain vasculature (a) before and (b) 45 min after maternal exposure to ethanol at a dose of 0.75  g/kg. The dashed rectangle shows the main branch of the vessel on which the quantifications were performed.

Vasculature images of one typical example from each of the groups are shown here. Figures 1–4 show MIPs of 3D cm-OCA images before and 45 min after gavage with distilled water (sham), and ethanol at doses of 3, 1.5, and 0.75  g/kg, respectively.

**Fig. 5:**
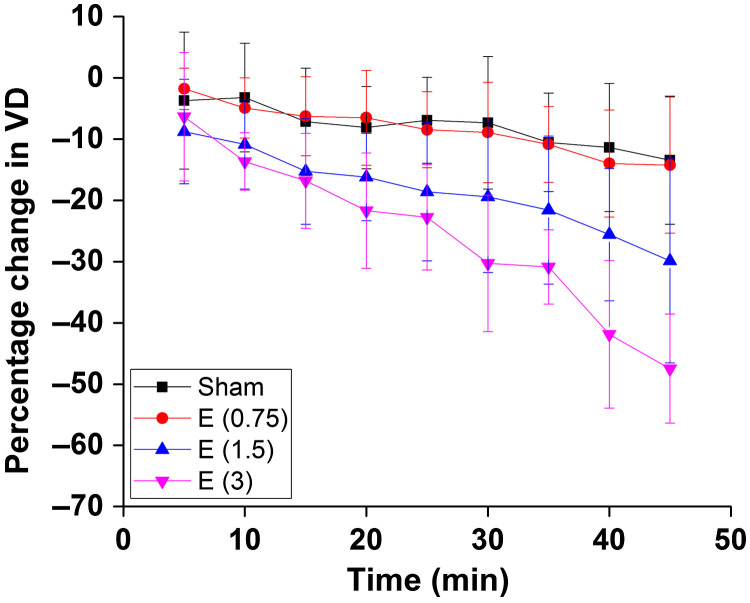
Percentage change in VD after exposure, every 5 min for 45 min. The error bars represent the standard deviation.

All three doses of ethanol showed dose-dependent vasoconstriction. Figure 5 shows the percentage change in VD for a period of 45 min at 5 min intervals where the pre-gavage measurement was used for comparison for that given dose. All samples from all four groups were used for these calculations. The plot depicts the inter-sample means and standard deviations. While the sham group and ethanol at a dose of 0.75  g/kg showed similar trends, the greater doses of 1.5 and 3  g/kg, ethanol exposure resulted in dose-dependent vasoconstriction.

The Kruskal–Wallis ANOVAs showed that the dose and time had statistically significant effects on the VD. The results of the Kruskal–Wallis ANOVAs are given in Table 1.

**Table 1:** Results of the Kruskal–Wallis ANOVAs. DF represents the degrees of freedom.

	DF	$\chi^{2}$ value	P value
Time	8	34.84	<0.001
Dose	3	54.45	<0.001

**Fig. 6:**
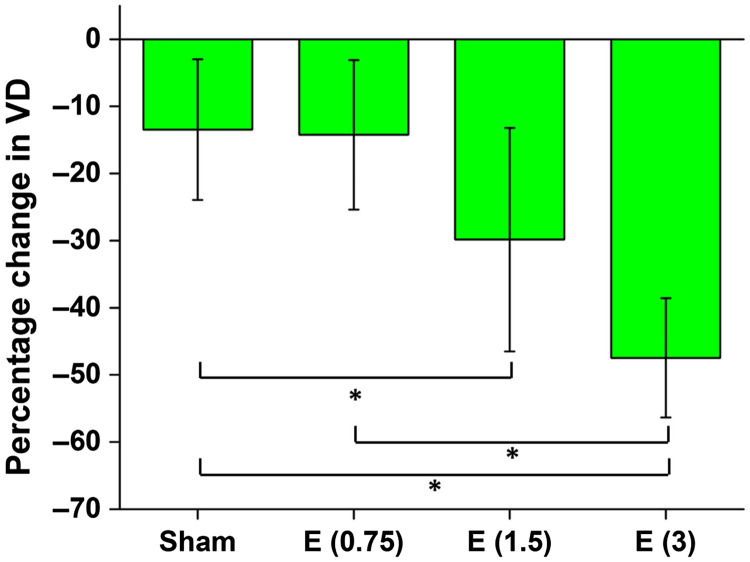
Percentage change in VD 45 min after maternal exposure to water (sham), and 16.6% ethanol at doses 0.75, 1.5, and 3  g/kg. The asterisk indicates P<0.008 using a two-sided Mann–Whitney U test.

Figure 6 shows the dose-dependent changes in the fetal brain VD 45 min after maternal exposure. The inter-sample averages and standard deviations are plotted. The results from the ethanol groups were compared to results from distilled water (sham) and to each other, and the statistically significant results (P<0.008 after Bonferonni correction) are shown by the asterisks.

Six two-sided Mann–Whitney U tests were performed between each of the groups for pair-wise testing. A statistically significant difference was found between the sham group and the 3  g/kg ethanol group, between the sham group and the 1.5  g/kg ethanol group, and between the 0.75 and 3  g/kg ethanol groups. Table 2 shows the results from the six pair-wise Mann–Whitney U tests. P values in bold indicate statistical significance after Bonferroni correction for multiple tests (P<0.008).

**Table 2:** Summary of the Mann–Whitney U tests. P values in bold indicate statistical significance (P<0.008 after Bonferroni correction for multiple tests). n1 and n2 are the number of samples in group 1 and 2 being compared. U is the test statistic, and P<0.008 indicates the criterion for statistical significance.

Test	n1	n2	U	p
Sham versus 0.75	18	15	132	0.9
Sham versus 1.5 g/kg	18	12	173.5	**0.005**
Sham versus 3 g/kg	18	9	162	$3.46\times 10^{-5}$
0.75 g/kg versus 1.5 g/kg	15	12	141	0.013
0.75 g/kg versus 3 g/kg	15	9	135	$6.44\times 10^{-5}$
1.5 g/kg versus 3 g/kg	12	9	86	0.025

### 3.2 Comparison of Acute Vasculature Changes in the Mother and the Fetus

**Fig. 7:**
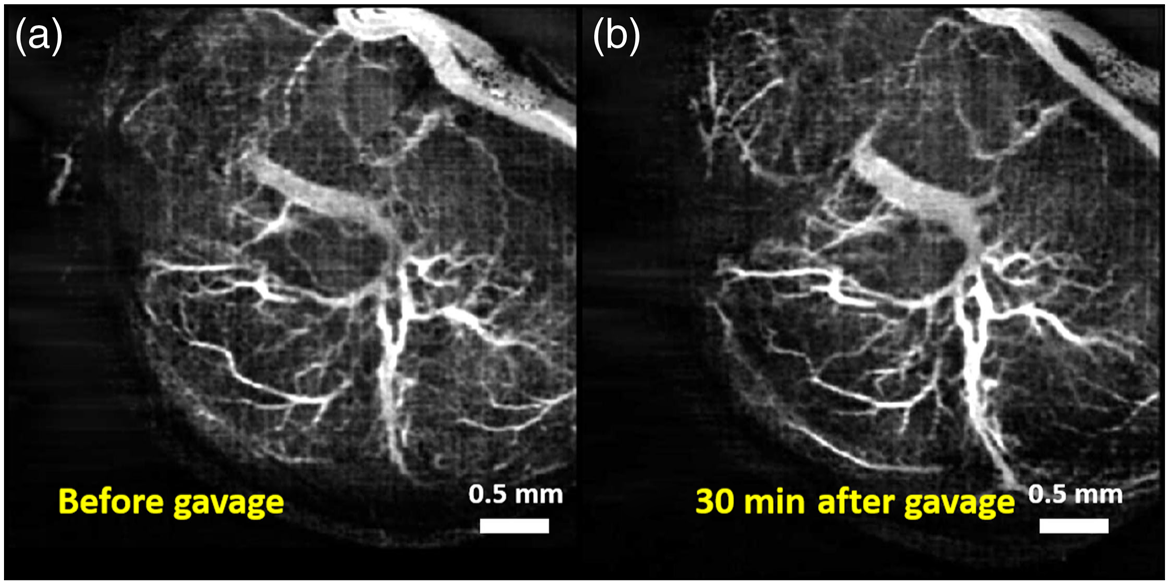
MIP of cm-OCA images of fetal brain vasculature (a) before and (b) 30 min after maternal exposure to lactated Ringer’s at a dose of 3  g/kg.

**Fig. 8:**
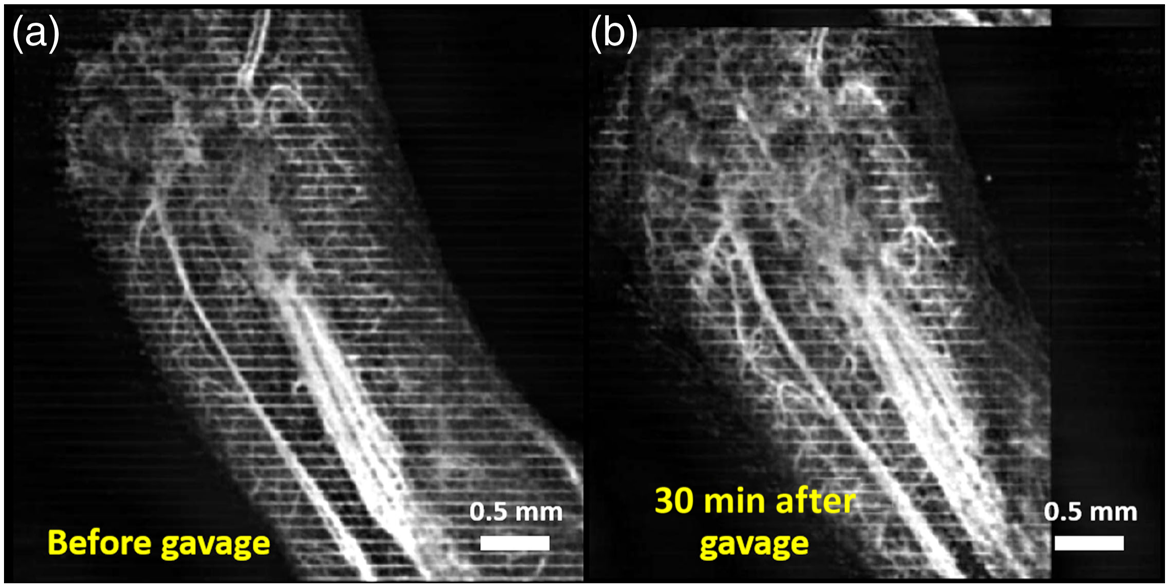
MIP of cm-OCA images of the hindlimb of the mother (a) before and (b) 45 min after maternal exposure to lactated Ringer’s at a dose of 3  g/kg.

Figures 7(a) and 7(b) show MIPs of 3D cm-OCA images of the fetal brain before and 30 min after exposure to lactated Ringer’s, respectively. Figures 8(a) and 8(b) show MIPs of 3D cm-OCA images of the mother’s skin on her hindlimb before and 30 min after exposure to lactated Ringer’s, respectively. No significant change in vasculature was seen in the results from the fetus and the mother.

**Fig. 9:**
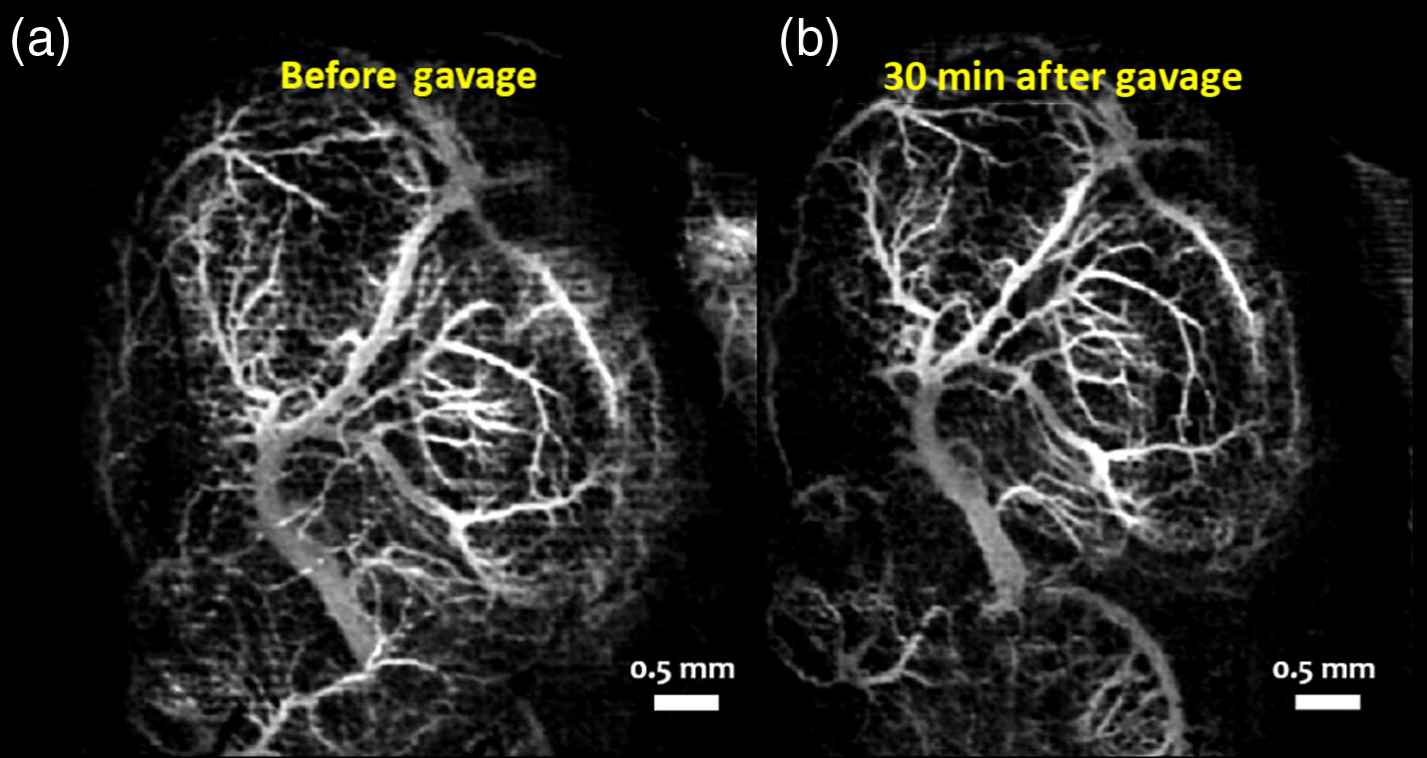
MIP of cm-OCA images of fetal brain vasculature (a) before and (b) 30 min after maternal exposure to ethanol.

Figures 9(a) and 9(b) show MIPs of 3D cm-OCA images of the fetal brain before and 30 min after exposure to ethanol, respectively. Similar to results shown in Figs. 2 and 3, a drastic vasoconstriction was seen in the fetal brain within 30 min of ethanol exposure.

**Fig. 10:**
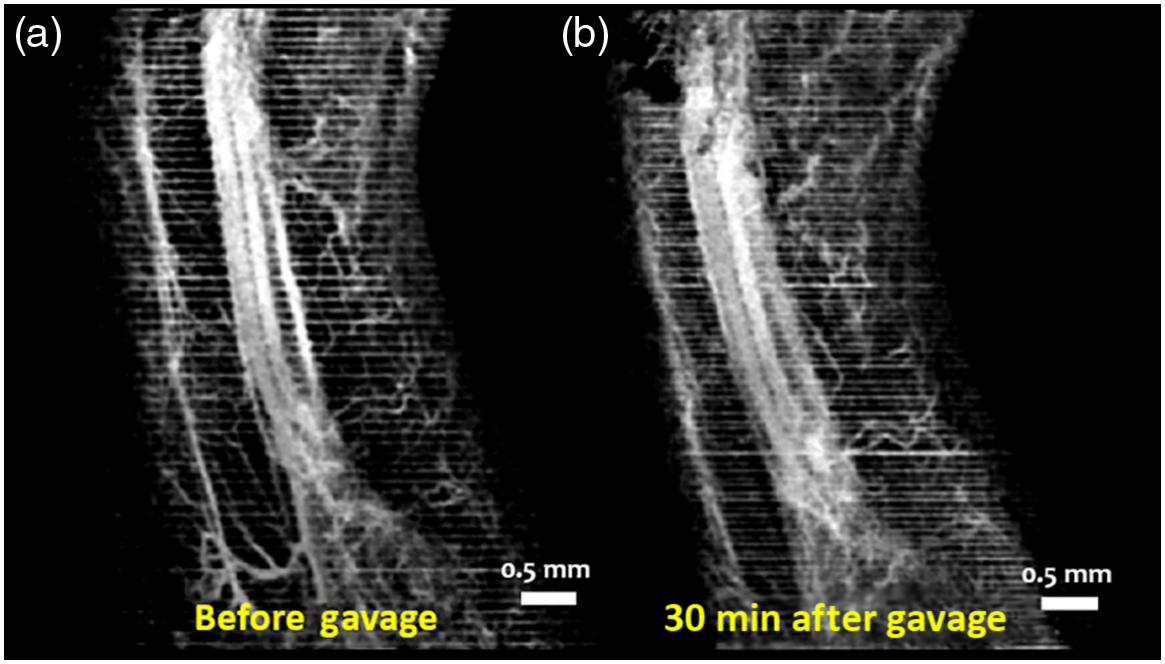
MIP of cm-OCA images of the hindlimb of the mother (a) before and (b) 45 min after maternal exposure to ethanol.

Figures 10(a) and 10(b) show MIPs of 3D cm-OCA images of the mother’s skin on her hindlimb before and 30 min after ethanol exposure, respectively. Drastic vasodilation was observed, which is in contrast to the vasoconstriction seen in the fetal brain. These results are similar to previous studies in humans where vasodilation was seen in forearm skin, whereas vasoconstriction was noticed in the underlying muscle.^52^

**Fig. 11:**
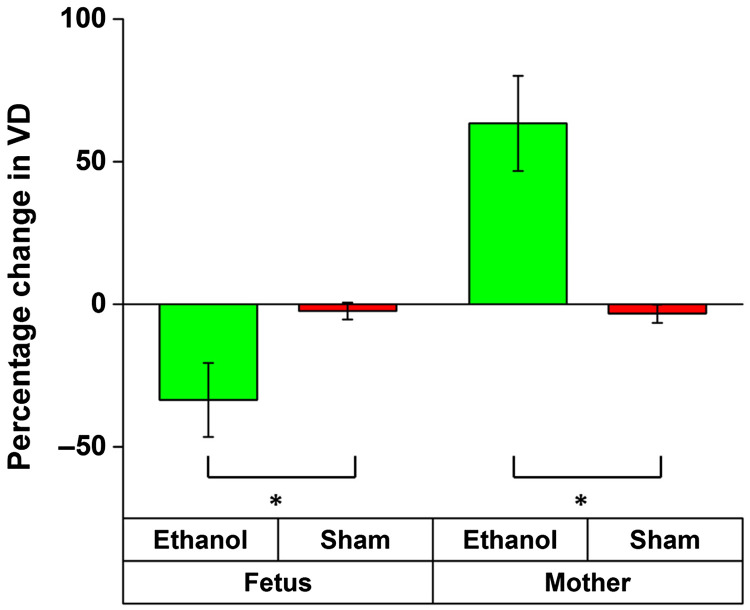
Comparison between percentage change in VD 30 min after exposure to ethanol and lactated Ringer’s. The asterisk indicates P<0.001 using a two-sided Mann–Whitney U test.

Figure 11 plots the comparison of percentage changes in VD at 45 min after exposure. The results from the ethanol groups were compared to results from the sham group from the mother and fetus, respectively. The inter-sample averages and standard deviations of percentage change in VD were plotted.

Results from Mann–Whitney U tests performed to compare results from the ethanol and sham samples in the mother and fetus groups, respectively, are shown in Table 3.

**Table 3:** Summary of the Mann–Whitney U tests. P values in bold indicate statistical significance. n1 and n2 are the number of samples in the ethanol and sham groups. U is the test statistic and P<0.05 indicates the criterion for statistical significance.

	n1	n2	U	p
Fetus	9	9	81	<0.001
Mother	9	9	0	<0.001

## 4 Discussion

Alcohol and a number of other teratogens inhibit CNS development, which is an effect that is often associated with inhibition of neurogenesis. However, since the neurogenic period is also a critical period of angiogenesis,^30^ neuro-vascular development may be equally drastically affected by teratogen exposure during this period of gestation. In this study, we assessed acute dose-dependent changes in the murine fetal brain vasculature after maternal exposure to ethanol. Two doses (0.75 and 1.5  g/kg), in addition to a dose reported in our earlier study (3  g/kg), were tested. Results showed an expected reduction in vasculature with higher doses, and overall, a reduction in vasoconstriction correlated with a reduction in the dose administered. In addition, this work also presented results comparing simultaneous vasculature changes in the peripheral vessels of the mother and the fetal brain vessels after maternal exposure to ethanol. Results showed a drastic reduction in fetal brain VD, whereas the peripheral vessels of the mother dilated.

Previous studies have shown that chronic alcohol exposure diminishes the relaxation of uterine artery in response to acetylcholine.^55^ However, the acute response of maternal blood vessels during pregnancy has not been assessed. Therefore, our data showing that maternal peripheral vessels undergo vasodilation whereas fetal cerebral vasculature undergoes acute vasoconstriction supports a hypothesis that acute alcohol exposure results in decreased blood pressure in the mother, which along with decreased blood flow to the fetal brain, is likely to significantly diminish nutrition supply during the critical window of fetal neurogenesis.

The decrease in VD observed in the sham group could be due to various factors such as prolonged exposure to isoflurane anesthesia or due to partial occlusion of the uterine vasculature while immobilizing the uterus and fetuses. However, since these factors are common procedures used in all samples, we can safely assume that the influence is similar in all samples and that any differences in results are due to the differences in administered drugs (or shams).

The previous study utilized a speckle variance algorithm to obtain vasculature maps,^47^ whereas this study utilized cm-OCA.^50^ Hence, the 3 g/kg dose data, as shown in Fig. 1, were reprocessed using the cm-OCA algorithm for consistent comparisons. Since the effects of immobilizing the uterus and anesthesia sometimes led to the disappearance of smaller tributaries, the main branch of the vessel was chosen for quantifications to ensure that no bias was introduced.

This study assessed acute changes in fetal brain vasculature and showed results for a period of 45 minutes only. To determine if the effects of maternal ethanol exposure are persistent we are presently conducting longitudinal studies. Our future studies will also ascertain if changes in the fetal and uterine blood flow, if any, are correlated. Our previous work has focused on acute exposures to individual teratogens.^47^^–^^49^ However, drug co-abuse is common^56^^,^^57^ and can occur with other risk factors, including maternal stress,^58^^,^^59^ cardiovascular,^60^ and metabolic disease.^61^ Hence, our future work will also involve studying these co-occurring factors and co-abuse of different drugs.

It should be noted that there are limitations to the current imaging technique. For example, system sensitivity, and sensitivity roll-off affects phase stability,^62^ which in turn affects the quality of the cm-OCA map. To improve the sensitivity, similar to all of our other studies, we ensured that the fetus was oriented in such a way that there was good visualization of the dorsal vessels in the brain. Our future work will involve a projection resolved algorithm^63^ to reduce the shadowing artifacts and to image deeper vessels. To enhance vessel contrast and connectivity, we also plan to use a phase correction scheme,^50^^,^^64^ a 2D Gabor wavelet filter,^65^ and faster imaging speeds to reduce the presence of bulk motion.

## 5 Conclusion

This study evaluated dose-dependent murine fetal brain vasculature changes due to maternal exposure to ethanol using *in utero* cm-OCA. Vasoconstriction was observed, and the extent of the effect reduced with a decrease in dose. In addition to this, results from simultaneous imaging of the mother’s peripheral vessels and the fetal brain vasculature to assess the effects of ethanol showed vasodilation in the mother and vasoconstriction in the fetus.
